# Addressing the fear and consequences of stigmatization - a necessary step towards making HAART accessible to women in Tanzania: a qualitative study

**DOI:** 10.1186/1742-6405-8-28

**Published:** 2011-08-02

**Authors:** Zahra P Theilgaard, Terese L Katzenstein, Mercy G Chiduo, Christiane Pahl, Ib C Bygbjerg, Jan Gerstoft, Martha M Lemnge, Britt P Tersbøl

**Affiliations:** 1 Department of Infectious Diseases, University Hospital of Copenhagen, Blegdamsvej 9, Copenhagen, DK-2100, Denmark; 2 National Institute for Medical Research, Tanga Centre, Bombo Road, Tanga, Tanzania; 3 Department of International Health, Immunology and Microbiology, Faculty of Health Sciences, University of Copenhagen, Øster Farimagsgade 5, Copenhagen, DK-1014, Denmark

## Abstract

**Background:**

Highly Active Antiretroviral Therapy (HAART) has been available free of charge in Tanga, Tanzania since 2005. However we have found that a high percentage of women referred from prevention of mother-to-child transmission services to the Care and Treatment Clinics (CTC) for HAART never registered at the CTCs. Few studies have focused on the motivating and deterring factors to presenting for HAART particularly in relation to women. This study seeks to remedy this gap in knowledge.

**Methodology:**

A qualitative approach using in-depth interviews and focus group discussions was chosen to understand these issues as perceived and interpreted by HIV infected women themselves.

**Results:**

The main deterrent to presenting for treatment appears to be fear of stigmatization including fear of ostracism from the community, divorce and financial distress. Participants indicated that individual counselling and interaction with other people living with HIV encourages women, who are disinclined to present for HAART, to do so, and that placing the entrance to the CTC so as to provide discrete access increases the accessibility of the clinic.

**Conclusion:**

Combating stigma in the community, although it is essential, will take time. Therefore necessary steps towards encouraging HIV infected women to seek treatment include reducing self-stigma, assisting them to form empowering relationships and to gain financial independence and emphasis by example of the beneficial effect of treatment for themselves and for their children. Furthermore ensuring a discrete location of the CTC can increase its perceived accessibility.

## Background

HAART has been available free of charge in Tanga, Tanzania since 2005. The treatment is provided and monitored at specialized HIV clinics called Care and Treatment Clinics (CTC). The CTCs also monitor and care for people living with HIV, who are not yet eligible for HAART. Prevention of mother-to-child transmission (PMTCT) of HIV is an integral part of antenatal care in Tanzania, and PMTCT services are centred at the Reproductive and Child Health (RCH) clinics. All pregnant women, who come to RCH clinics for care, are thus counselled about and tested for HIV, unless they opt out. HIV infected women, whose CD4 count is > 200 cells/μl, are provided with short-course ART for PMTCT. Those, whose CD4 count is < 200 cells/μl, are referred to the CTC to start life-long HAART [1].

Since 2006 we have been conducting a PMTCT study in Tanga, the ComTru Study [2]. The study has referred women, who were entitled to HAART, to the CTC at Bombo Regional Hospital (BRH) since 2006, and to the CTCs at Ngamiani Health Centre and Makorora Health Centre since these were opened in 2009. Study participants are referred to a CTC postpartum for continued monitoring and treatment. In February 2008 we investigated how many of the women, who had been referred from the ComTru Study since its beginning, could be traced at BRH CTC. We found that more than 60% had never reported to the CTC, and only 10% were still affiliated to the CTC 12 to 18 months after referral, even though the RCH, from where they were referred, and the CTC are situated on the same hospital premises [3]. Subsequently, efforts to encourage referred women to report to the CTC were increased, including extended post-test counselling and escort to the clinic. Despite these efforts more than 35% of the patients, who had been referred from February 2008 to February 2009, had not been registered at the CTC by mid-March 2009 [4]. Similarly, studies done by the Tanzanian National AIDS Control Program (NACP) from 2005 to 2007 show that the percentage of patients on HAART, who collect their drugs every month, falls to 34% after six months treatment, to 25% after one year and to 10% after two years [5].

Among the primary reasons for non-adherence to HAART programmes found in previous qualitative studies from Sub-Saharan Africa, were transport costs or lack of means of transport, distance to clinic, long waiting times and loss of income [6-9]. Other reasons unearthed through these studies were lack of adequate food, fear of stigma, having to take the medicines covertly in order to hide their status, side effects, poor counselling from overworked staff, perceived lack of support from family and peers' scepticism about the effect of the treatment, repeated registration fees and cost of treatment of opportunistic infections [6,7,9]. Studies from a rural area in Tanzania reported that major determinants of clinic attendance included perceived disease susceptibility and severity, misconceptions about HIV aetiology and the effect of ART and support received from friends and family [10,11]. All the above mentioned studies have included both genders. The only study found to have investigated barriers to accessing HAART particularly for women was a study of the barriers to accessing a PMTCT plus programme in Uganda [12]. This study also found that transportation costs were the main barrier, but that financial dependence on partners was an exacerbating factor [12]. Other deterring factors were fear of stigma, lack of disclosure, fear of being exposed as HIV infected, long waiting time at the clinic and negative interaction with the staff [12]. The presence and importance of each of the deterrent factors varied from setting to setting and was naturally influenced by the population studied and the context.

It is commonly acknowledged that women are at higher risk of HIV infection in Sub Saharan Africa. In Tanzania, in the age group 15-24 years, women are four times more likely to be infected with HIV than their male counterparts [13]. The higher risk of HIV infection can be attributed to gender-based differences: Women's lower social and economic status compromises their ability to make healthy and safe decisions about their own lives including decisions about sexual debut, while men use their higher social and economic status to impose their will [14]. Furthermore economic need encourages women to engage in transactional sex. Thus 42% of females aged 18-25 years interviewed at a Sexually Transmitted Infections (STI) clinic in Dar es Salaam had exchanged sexual favours for gifts or money [15]. This difference in autonomy that is related to gender makes a study of women's perception of the accessibility of the HAART programme particularly relevant.

The expansion of the HAART programme in Tanga to include distribution at primary health centres has brought the treatment closer to the patients. We hypothesized that barriers other than transport related issues were behind the low uptake of and adherence to HAART found among women in this setting [3]. The expansion of the PMTCT programme has made the RCH clinics one of the most common testing sites, and hence one of the most common sites from which women are referred for care and treatment at the CTC. Consequently identifying potential challenges to successful referral of HIV infected women from PMTCT services to the CTC is vital. Therefore the current study explored factors relating to referral of women diagnosed with HIV in conjunction with PMTCT services from the RCH to the CTC.

HAART is socially as well as pharmaceutically active; taking or not taking HAART has social causes and consequences that may only really be explored by examining, in context, patients' experiences and perceptions from a qualitative point of view [16]. Therefore a qualitative approach was chosen to understand HIV positive women's perspectives and their perception of the HIV related services and options available to them [17,18]. The data collection techniques employed were participant observation, in-depth interviews (IDI) and focus group discussions (FGD).

### Study Setting

Field work for this study took place from December 2009 to April 2010 in Tanga, Northeastern Tanzania, one of the largest cities in Tanzania with a population of approximately 240000. The main sources of income are agriculture, livestock keeping and fishing as well as various small businesses including handicrafts, fishmongery, small shops and food stalls [19]. The HIV prevalence in Tanga Region in 2007-2008 was 4.8% [20]. The provision of HAART is being scaled up to include distribution and monitoring at CTCs at health centres in addition to the distribution that takes place at Bombo Regional Hospital [21]. Thus CTCs were opened at Makorora and Ngamiani Health Centres in February 2009.

In 2009 the FAUMA Club (acronym made from the words FAraja meaning comfort, Upendo meaning love and MAtumaini meaning hope) was established by the local NGO that provides voluntary counselling and testing and home based care for people living with HIV PLHIV, called Tanga AIDS Working Group (TAWG), and nurses and researchers from the ComTru Study. The Club was established following reports of frustrations related to disclosure of HIV status and how to live positively after receiving their HIV diagnosis by women newly diagnosed with HIV at the RCH. The aim of the club is to be a forum for HIV-infected pregnant women and mothers, who meet to discuss issues and receive education related to their HIV-infection.

## Methodology

### Field work

The first author, ZPT, spent two years in Tanga as project coordinator of the ComTru Study and as one of the coordinators of the FAUMA club. Working in the study area for an extended period of time gives particular insight into the general context that forms the experiences of the study population and a background against which the content of interviews and focus group discussions should be interpreted.

The longer-term presence allowed for participant observation as during this time ZPT participated in patient consultations at the RCH and in FAUMA club meetings to observe her study populations and their interaction. Observations during these events have also assisted a more contextually grounded analysis of the data.

In-depth interviews (IDI) were chosen as a technique, because they are similar to day to day conversations, thus encouraging the respondent to feel at ease. The flexibility of this technique allows the respondent to emphasize what she feels is important, instead of having to fit her views and interpretations into categories predefined by the researcher. It is possible to ask clarifying questions and to further probe into perspectives introduced by the respondents, which enables complex and sensitive issues to be explored [17,22]. As the focus of the research was clearly defined, a semi-structured interview format, with a written interview guide was chosen [23]. Focus groups discussions (FGD) were chosen to allow the researcher to see how the participants assessed the topics in a group forum, where they can challenge each other's views as well as elaborate or agree on statements made by others [24]. Once again a semi-structured format, with a written discussion guide was chosen.

The study population contained four distinct groups:

*Group 1: Fauma Club members*

The first group comprised 12 women from the FAUMA Club, sampled from a larger group of volunteers in order to get the perspectives of self- assured vociferous women as well as those of more quiet women. One FGD took place within this group and lasted about 80 minutes. The FGD was held on a Saturday afternoon in the same Primary School Social Hall where the FAUMA Club usually convenes, in order to ensure a familiar and private setting.

*Group 2: ComTru (CoT)Study women*

The second group consisted of 24 HIV infected women recruited from the RCH Clinics via the ComTru Study. These women participated in one or two IDIs. At the time of the first IDI six of the women were pregnant and 18 of them had given birth during the previous 18 months. The women, who were pregnant, were being referred to the CTC, because their low CD4 count entitled them to HAART. The women, who were interviewed after having given birth, had participated in the ComTru Study and had been monitored by the ComTru Study team for at least nine months post-partum at the time of their first IDI. The second IDI was held approximately three months after referral to the CTC. Fourteen of the initial 24 women chose to participate in this interview - five of the initially pregnant women, and nine of the women who had already given birth. The duration of the interviews varied with a maximum of 80 minutes and they took place upon the participants' request either in a quiet room at the RCH clinic where they were recruited or at a different clinic.

*Group 3: CTC patients*

The third group included four women recruited directly from the CTCs, as we wanted to learn how women, who have not been referred from RCH clinic services and in particular not from the ComTru Study, experience the CTC and taking ART. One interview was held with each of these women. The interviews took place in a secluded room at the CTC or at the RCH clinic, and lasted from 30 minutes to 58 minutes.

*Group 4: TAWG and RC Counsellors*

The fourth group was composed of three counsellors and a clinician from TAWG and two home based care workers from the Red Cross (RC). This group was chosen in order to obtain information about how HIV infected women's situation is perceived by people who work closely with them, who are very familiar with the CTCs, but who do not work there themselves. The discussion took place in TAWG's meeting room.

The interviews were primarily conducted in Kiswahili by ZPT or the fourth author, CP, with assistance from a translator. The FAUMA Club FGD was likewise conducted in Kiswahili, and was facilitated by and translated to ZPT by the third author, MC. The TAWG and RC Counsellor FGD was conducted in a mixture of Kiswahili and English by ZPT with translation assistance provided by the participants themselves. All IDIs and FGDs were recorded, transcribed and translated prior to data analysis. The quality of the transcription was spot-checked by ZPT, and the quality of the translations was checked with assistance from MC.

### Ethical considerations

The study was approved by the Medical Research Coordination Committee of the National Institute for Medical Research of Tanzania (Reference number NIMR/HQ/R.8a/Vol. IX/893). Participants were provided with oral and written information about the study and gave written consent to participate and to be quoted. Anonymity of the study participants has been ensured through giving each interview participant a code and study staff was carefully instructed to keep the confidentiality of the participants. Interviews were conducted in a respectful manner and participants were encouraged to ask questions and to interrupt the interview at any time. At the completion of interviews participants were again encouraged to raise any questions or concerns that they might have, which often lead to AK and ZPT giving a short counselling session.

### Data Analysis

Domain analysis as described by Atkinson and Abu El Haj [25] was used to analyse the content of the interview and focus group discussion data. Based on extensive study of the transcribed interviews and FGD, the following 4 consecutive steps were followed:

1. The first step was identification of domains, which are the main areas addressed by research participants in the data.

2. The second step was to construct a taxonomy of the subcategories that emerge from the data. The subcategories were identified directly from the data, and the taxonomy was continuously adapted as information emerged.

3. The third step was to summarize what the participants have said about the subcategories into key themes that must be addressed.

4. Identifying relations between the sub-categories in order to build up an overall picture.

The key themes identified in the study were 1. Diagnosis and disclosure; the fear and reality of repercussions, 2. Deterrents from seeking HAART and 3. Return to being a normal person; sources of encouragement and motivation to seek HAART.

A summary was then prepared by further grouping of the data and finally a mind map was made to establish relations between the various domains and their subcategories.

### Quality of the data

Lincoln and Guba have defined the credibility of qualitative data as the naturalists' (or qualitative researchers') internal validity. They have described three essential elements to increase the credibility of qualitative research [26]. They are 1. prolonged engagement in the field, which enables the researcher to learn about the context, minimize distortions and build trust, 2. persistent observation and 3. triangulation [26].

#### Prolonged engagement, persistent observation and building trust

Due to the duration of ZPTs stay in Tanga and her involvement with the FAUMA club, the women who participated in the FAUMA FGD had become familiar with ZPT prior to the FGD.

The women in the IDI were interviewed in Kiswahili by ZPT or the fourth author (CP) with assistance from one of the ComTru Study nurses (AK). AK was chosen as a translator because of her natural ability to make participants feel at ease and trust her, as was mentioned by several participants in the interviews. Before beginning the interview time was taken to inform the participants in detail of the nature of the study, including how the information obtained would be used, and to assure the participants that their identities would remain concealed. Instead of approaching the subject of HIV directly, the participants were encouraged to talk about their backgrounds, families and socio economic circumstances first.

The fact that the interviewer and translator were both health care personnel led us to consider whether some women would not be honest about their experiences with health care personnel, the PMTCT services and the CTC. However, the women disclosed negative experiences with CTC personnel and criticized conditions at the CTC. In addition, intimate issues such as fear of abandonment, discrimination and challenges in their relationships were raised by the participants, which lead us to believe that those, who voiced these concerns, felt comfortable enough to trust us and share their experiences.

#### Triangulation

Our approach to data collection ensured that we could compare interview data with FGD data and our participant observations made at clinics and during Fauma club meetings. Themes emphasized by the women in the interviews were compared with the information provided by the TAWG/RC counselors and by the women in the FAUMA club. Thus we assessed whether HIV infected women provided the same conclusions when the information was solicited through IDI and FGD. We also assessed whether the information provided by a different category of respondents, i.e. the counselors, supported the information provided by the women. The considerable overlap in themes brought forward by the counselors and the HIV infected women consolidates the findings. Furthermore insight that has emerged from the IDI and FGD correspond to observations made during informal visits to the clinics.

#### Limitations

This study faced 2 major challenges during data collection. The TAWG and RC FGD was a success as the six participants fed off each other's answers and discussed the issues at hand. However the FAUMA FGD was attended by 12 women, which proved to be too large a group. The session did not turn into a discussion as the women did not contradict each other or feed off one another's answers. Rather they tended to agree with each other All women contributed to the discussion at least once, however there were some women who were more shy and reticent than others. The participants who dominated the conversation were women who had known their status for some time, and who had positiv experiences with the CTC. Thus not enough effort was made to solicit information about barriers to going to the CTC during this session. When we compare to data from IDI and our observation, the lack of discussion may also stem from the fact that the women have very similar experiences and therefore didn't disagree with each other. The women did provide substantial information, including personal experiences of being abandoned by their husbands after having disclosed their HIV status. Therefore we believe that the data from the FAUMA FGD is valid.

The second challenge was that of the 24 patients from the ComTru Study who were interviewed initially, only 14 could be persuaded to return for their second interview. Of the 10 patients, who did not return for their second interview, four had not reported to the CTC since referral. Thus valuable information about barriers was not obtained from them. We do not have information about why they chose not to return. However we can hypothesize that some of the concerns about going to CTC may have also dissuaded them from returning for the second interview. Fear of whether their statements would truly remain anonymous and fear of being identified as HIV positive through participation in the interview may have deterred them from returning for their second interview. These fears may have been magnified by the use of the dictaphone. It was our impression that the dictaphone did in some cases cause shyness initially, but that the shyness was overcome as the interview progressed as seen by personal and sensitive information provided during the interviews.

## Results

### Study population

The FAUMA FGD participants were 22 to 38 years of age (mean 31.5 years). Seven women described themselves as married, four as single or divorced and one as widowed.

Baseline characteristics were available for 23 out of the 24 participants recruited for IDI from the ComTru Study. Only marital status was available from the last woman. Their age ranged from 21 to 43 years (mean 27 years). Fifteen were married, four were cohabiting with their partner, one was divorced, one was separated and three were single. Twenty were Muslim and three were Christian. Seventeen had primary education and six had secondary.

Baseline characteristics were not collected systematically from participants recruited directly from the CTC. However the interviews indicate that the situations and concerns that this group of women shared with us were in accordance with those of general study population.

### Main themes

Three main themes emerged from the data: 1. Diagnosis and disclosure; the fear and reality of repercussions, 2. Deterrents from seeking HAART and 3. Return to being a normal person; sources of encouragement and motivation to seek HAART. Analysis of the data showed that stigmatization toward HIV patients, although not ubiquitous, is still highly prevalent in Tanga and incurs a fear of ostracism from the community, divorce and financial distress in women diagnosed with HIV. The other fear faced by HIV infected pregnant women and mothers is that they will not be able to take care of their children. The fear of ostracism, divorce and financial distress is a weighty deterrent from seeking life prolonging HAART at the CTC, while the fear of not being able to take care of their children is among the strongest of motivators.

### Diagnosis and disclosure; the fear and reality of repercussions

"*If they find out they stigmatize you, they see you as a strange person. They don't see you as a normal human being - in general they stigmatize you*." CoT IDI 17

This statement is representative of the description given by the majority of the participants in this study, when asked how PLHIV are perceived in Tanga. The stigmatization is expressed through actions ranging from gossiping and finger-pointing through exclusion from social interaction such as sharing a meal to complete segregation.

Shock, confusion, worry, fear, sadness, despair and depression are words used by the participants to describe how they felt immediately after receiving their diagnosis.

"*I was out of my mind, confused, shocked, astonished - Why should I suffer so much?*" CoT IDI 13

"*I thought it was the end of my life. All the plans I had vanished. I started to despair*." CoT IDI 23

Because of fear of stigmatization and of becoming the subject of malicious gossip seeking comfort from friends and relatives is not an evident course of action.

"*They will segregate me because I am infected. (...) That is why I remain silent. If anyone discovers it fine, but I can't go and announce it to everyone, because we Swahili people if you quarrel (with someone) she/he will announce that you are infected! Everyone will despise you! They will be afraid of you and they won't ask for water from you*." CoT IDI 6

The counsellors confirmed the reality of this stigmatization.

"*And another thing is a belief out there that, 'he, who tests first, is the one who is infected.' So she is afraid of telling her husband, because he will tell her that she is the source (of the infection)(...). And the family members, like brothers and mother they believe that every person who is infected, he/she got it through sex, so they are afraid of being called prostitutes*." TAWG/RC FGD

Disclosure to their partner can be particularly problematic. Most of the women interviewed only had primary education and one third of them were entirely financially dependent on their partner. Several women said, they had given up working because of their pregnancy, or because they have a small child. The anticipation of abandonment, violence and withdrawal of financial support for themselves and their children prompts the women to keep their status a secret from their partner.

*"He has sworn to kill me several times and he is abusing me daily. He is not a person to advise these kinds of things (meaning: he is not a person to whom she can disclose her status) because I will be looking for trouble." *CoT IDI 5

"He hasn't discovered (that I have HIV) yet. (...) The way I see their procedures even if I will tell him, it means they (her husband and his family) will torture me. I won't have fare (money) to return back home." CoT IDI 15

The fear of abandonment by their partner is well founded, as some of the women who chose to disclose have experienced.

*"After those problems we separated. Yes, he dumped me because I am infected." *FAUMA FGD

*"I informed my husband and he went to test and discovered that he is not infected. He divorced me and said he can't live with some one of my kind." *FAUMA FGD

This picture was also corroborated by the counsellors, who postulated the fear of the financial repercussions of divorce as a reason for non-disclosure of HIV status by women. In contrast to this, they maintain that HIV infected men do not face the same risk of abandonment by their wives.

*Most of the time men become so sad when they are discovered to be HIV positive and their wife is discovered to be HIV negative, and as it is in the women's character they feel sympathy and pity over the men (...)They take into consideration that this is my husband I have already taken an oath (of marriage)*. TAWG/RC FGD

Furthermore, pregnant women are strongly encouraged to be tested for HIV for PMTCT. Thus a woman's HIV status is often established before that of their male partner. Men have the prerogative to refuse to be tested, and often do so, thereby protecting themselves from stigmatization.

I told him do you know I went for the test 2 times,(...) please could you go for the test. He told me I won't go because I'm not affected. CoT IDI 7

Thus the perception of PLHIV in the community, which may lead to potential abandonment and socio-economic deprivation, combined with opt-out testing for HIV being part of antenatal care, puts pregnant women at risk of finding themselves in a uniquely precarious situation.

### Deterrents from seeking HAART

The CTCs are only for PLHIV. Consequently, going there means exposing your HIV status and putting yourself at risk of stigmatization by the community. This is described as the main barrier to presenting for treatment - particularly if the woman does not yet perceive herself as sick.

*"I haven't gone to register myself at the CTC. I haven't gone to register myself because I haven't been sick. When you mention the issue of going to CTC my heart gets some sort of shock and I feel unhappy, depressed and different. That CTC place is for embarrassment and degrading yourself, because if you happen to be seen there by two or three people, they will no longer respect you and your status will be lowered." *CoT IDI 5

The layout of the CTC, and in particular location of the entrance in an indiscrete area, where other people at the health facility can observe who enters the CTC, decreases the accessibility as it increases the risk of stigmatization by the community.

"I don't like the venue, because the entrance is in a place where all the patients who come to the hospital line up to register. Therefore when you go there (to the entrance of the CTC), all the patients, who are present, will see you." CoT IDI 4

People seeking treatment at the CTC must bring an adherence partner in order to initiate treatment. An adherence partner is a person chosen by the patient, who is supposed to encourage the patient to adhere to the treatment. Identifying an adherence partner can however be problematic, because it requires disclosure. Thus disclosure is necessary in order to obtain treatment. As described above, disclosure means exposing one self and risking stigmatization and rejection. This may mean that in their desperation patients try to persuade someone, whom they do not know.

"*You can't start to give a patient medicine alone without her relative present, but some may say "I don't want my relative to know because of discrimination within the family". Therefore a person could suffer almost a month without starting to use medicine and she ends up by taking a person from the street, to be her witness in order for her to get medicine. But after few days people disappear because they chose someone from street and not their relative*." CTC IDI 2

The CTCs are only open for adults in the morning and early afternoon during the week. Therefore the frequent visits to the CTC required in order to receive HAART and adhere to the treatment programme combined with long waiting time often results in repeated, lengthy absence from work or other income generating activities. This may lead to loss of income in two ways. The first is the loss of profit from small business activities such as selling cooked food. The second is loss of employment due to absence from work or stigmatization by the employer. These factors cause PLHIV to refrain from seeking treatment.

"*Since I have been getting infections I waste my time coming here. At work, you always have to get permission that you will be late or you won't come. And sometimes when you get here to CTC, there are a lot of patients and only one doctor. (...) So you are supposed to follow the line (meaning wait in line), while at work they are waiting for you. It becomes a real problem*." CoT IDI 4

In a personal communication to ZPT some time after the interview this participant disclosed that she had been fired from her job due to absence for CTC visits.

The TAWG and RC counselors also emphasized that frustrations with poor service and management of tests at CTC and the cost of frequent futile visits discourages patients from going to the CTC.

The traditional healers are often the first people from whom treatment is sought for any kind of ailment. Therefore when PLHIV seek a traditional healer, they know what to expect.

*"Because most Africans used to go to the traditional doctors when they are sick, so that is normal. Not necessarily a specific kind of problems like sickness only, also some social stuff like she has a quarrel with her husband, or (problems with) business, she goes to the traditional doctor perhaps she had been cursed. So when you enter into the traditional doctor's house you can't be shocked by what happens there." *TAWG/RC FGD

As described by both patients and counsellors, there are a number of traditional healers in the community in and around Tanga, who claim to be able to cure HIV. Some PLHIV therefore choose to seek this treatment rather than the option provided by the CTCs, which requires taking medicine every day for the rest of their lives.

*"(Some people go to) the traditional doctors and get medicines, and those viruses die. I mean I just heard it from the place where I am doing my business of selling fish. There is a man who gives out medicines for AIDS. If you get 3 bottles of 5 liters all the viruses will die. So some of them (other PLHIV) are going there! Because they don't want to take drugs their whole life*." CoT IDI 6

Furthermore, traditional healers are described as less likely than health care workers at the clinics to disclose their patients' sero-status to others.

"*Usually the characteristic of traditional healers is that they are very secretive - they can take care of the patient without telling anybody. Some of the medical doctors they have that problem. Some of the doctors will say "you see that woman - she is living with HIV and still she is so strong!" So there is a tendency of medical doctors saying that, and also other medical professionals*." TAWG/RC FGD

### Return to Being a Normal Person: Sources of encouragement and motivation to seek HAART

Despite their fears most of the participants chose to disclose their HIV status to someone, and most received a supportive response, which lead to relief and to feeling once again like a normal person.

"*I was stressed the first weeks. After my husband comforted me I started to see it as a normal situation. First my husband advised me that I should not have stresses or be short tempered; I should live like a normal person, and eliminate this problem from my heart. I mean I should be like a normal person and I should eat to the fullest*" CoT IDI 17

"For sure I didn't feel good. Firstly it was my first time to give birth, then I didn't know what condition would happen to my kid to whom I will give birth, therefore after my mother comforted me I recovered my normal situation and I take it as if it is malaria despite that malaria has cure." CoT IDI 12

The importance and impact of counselling was highlighted by the participants and gave some of them the confidence to go to the CTC. Women, who were afraid to disclose their status to their partners and own social network, regained confidence through positive interaction with the nurses and doctors.

"*After they taught me and motivate me I have come to see myself as a normal person. At first I had some thoughts, but after what Mama X (one of the PMTCT nurses - her name has been removed to protect her privacy) taught me they have gone*" CoT IDI 24

*"No now I don't have at all (concerns about what people she met at the CTC are thinking about her) Due to that advice, every time when I consider that advice which they gave me, therefore I see it like a normal thing*. CoT IDI 9

When the participants were asked how to motivate those, who are afraid to attend the CTC, the importance of counseling was once again emphasized.

"*What to do is to find them, to talk to them on the fact of their condition, is not dangerous disease to fear, is like malaria (...) perhaps the classes they have been given is not going to their minds, they need to sit down and have a real education individually so as they could understand and be adherent*" CoT IDI 12

Interaction with other PLHIV helped participants to accept their HIV status and encouraged them to seek treatment.

"*I met with other people so I thought it is a global issue. I saw it as a problem which can happen to anyone. I met with children who are one year old at the CTC, elder women with their white hair and stick come to CTC! I realized that this problem can happen to anyone*" CoT IDI 6

Taking HAART is understood by the HIV infected mothers as the way to remain healthy and able to raise their children - an instinct which is a strong motivator.

"*Because I like to take care of my health and I have the dream to raise my child until he/she will help him/herself, therefore it's very important for me to check my health. That is very big issue which encourages me even today*" CoT IDI 8

Furthermore patients on HAART reported satisfaction with the effect of the drugs.

"I have taken ARVs, but they didn't disturb me. (...) At that time when I hadn't started using them yet I was very weak! I was weak and tired! I felt bad! But since I started taking medicines my strength is coming that is why I focus on taking the drugs" CoT IDI 1

Several of the women in the FAUMA FGD had been attending the CTC for some time, and expressed satisfaction with the services received there, including how they are treated by the staff.

*"I will be in Bombo (CTC) all days because, it is good, there is peace, there are good teachings, and if you are in trouble they help you without any problem." *FAUMA/RC FGD

These sentiments were echoed by IDI participants

"They are great, the nurses and doctors offer good treatment/services. There is no problem. Even before the date (to collect her medicine) I am around and am going there to get some advice where by it helps me a lot(...). " CoT IDI 4

## Discussion

Successful referral of patients from RCH services to CTCs requires that the motivational factors outweigh the deterrents. The main deterrent from seeking treatment identified in this study was fear of stigmatization leading to rejection by family and community and the loss of income. This section will therefore focus on these aspects and discuss possible solutions.

It is clear that being HIV infected is still a highly stigmatizing condition in Tanga, as it has been found to be in other parts of Tanzania [10,27] and Uganda [12]. In *Stigma, Notes on the Management of Spoiled Identity *[28] Goffman describes a person's social identity as her character and attributes as seen by those she comes into contact with. He defines stigma as "an attribute that is deeply discrediting". The bearer of such an attribute may be reduced in the minds of people from a "whole and usual person to a tainted and discounted one"[28]. The women interviewed in this study echo these definitions, as they describe their fear and experiences of not being regarded as normal human beings. Those, who found social support and trust in a new social network among other PLHIV, emphasized the notion of having regained a form of "normality".

Stigmatization emerged as a major threat to uptake of and adherence to life-saving HAART therapy, and must therefore be addressed. However, there is also an element of self-stigmatization: receiving the diagnosis "HIV infected" may change women's perception of themselves from normal to abnormal and hence not acceptable. This self-stigmatization may also lead to fear of disclosure to family and friends. Furthermore, fear of stigmatization can cause PLHIV to defer seeking treatment at the CTC until their HIV infection becomes so apparent that it can no longer be concealed, even when a low CD4 count has been found [10]. The fear may cause them to refrain from seeking treatment at the CTC altogether. Because of the fear of stigmatization, the location of the entrance to the CTC played an important role in how accessible the CTC was perceived to be. Location of the CTC and particularly the entrance to it in a discrete area was emphasized as one of the determinants when choosing which CTC to attend.

Divorce and loss of financial support were found to be possible outcomes of women's disclosure to their partners in this study as well as in a study from Dar Es Salaam [29]. This outcome of disclosure was described as unlikely to happen to male HIV infected individuals. Anticipation of abuse, abandonment and loss of financial support can lead women to withhold their status from their partner. This was also found in a similar study conducted in Uganda where lack of disclosure was the second most cited barrier to enrolling for treatment [12].

The main remedy suggested by participants to encourage PLHIV to go to the CTC was extended individual counselling. Statements made by participants in this study indicate that a close relationship with a counsellor can replace support that has not been solicited due to fear or has not been forthcoming from their partner or other family members. The effect of a trusted alternative social network was also found in Mwanza, Tanzania, where home-based care providers assumed the role of social network for patients lacking support [11].

The shortage of nurses in Tanzania means that nurses' capacities are often overstretched. Demanding that PMTCT nurses spend more time counselling and following up patients in the community is not realistic. Therefore, this gap must be filled in other ways. Alternatives that have been shown to have a beneficial effect in voluntary counselling and testing settings and HIV clinics are home based care providers [30] and peer health workers [31], often organized by non-governmental organizations exemplified by TAWG and the Red Cross in Tanga. Making their services part of the PMTCT care package could present a way to provide the necessary support services without further overburdening the RCH nursing staff. Another model could be to reemploy retired PMTCT nurses in the RCH clinics to carry out post-test counselling and continue to counsel participants as was done in the ComTru Study. Reemploying retired nurses to care for HIV patients has already been implemented successfully in Morogoro [32].

Meeting other PLHIV markedly improved the participants' perception of themselves. This impact was also emphasized by Roura et al [11], who found that the level of support received from post-test club members and other CTC clients contributed to retaining participants within the treatment programme, as did observing the positive effect of HAART on others for themselves [11]. Ensuring the opportunity for newly diagnosed HIV infected women to meet with other PLHIV in a context other than the CTC, for example post-test clubs for HIV infected women, would provide a social network where PLHIV feel they belong [33] and other benefits of such an encounter without the fear of stigma looming over it.

Contrary to findings from other studies [6-8,12] the economic concerns related to going to the CTC did not revolve around the cost of transport to the clinic. It seems this issue has been dealt with efficiently through the opening of CTCs at health centres. However proximity to the clinic does not in itself equate approachability. The necessity of repeated, lengthy absence from work due to the substantial time spent at the CTC in order to get treatment or having to come back time and again for CD4 counts was emphasized as a deterrent in two ways: PLHIV who are employed by others have to explain their absence, thus risking exposure of their HIV status, stigmatization and potentially losing their job. PLHIV who are self-employed lose their income, when they are not taking care of their business. Changing clinic opening hours to include some late afternoons and weekends was suggested as a logical course of action by participants in this study and others [7].

Finally, the potential loss of income resulting from disclosure to partner or exposure of HIV status at work must also be addressed. Some studies have been undertaken to investigate the potential of providing microcredit loans to PLHIV with promising results, including reports of women feeling more autonomous and more accepted as a result of receiving the loans [34]. Initiatives to promote financial autonomy for women living with HIV should be explored further.

## Conclusion and recommendations

This study has shown that that stigmatization of HIV infected women is still widespread in Tanga and that fear of the consequences of stigma is a major threat to uptake of HAART by HIV infected women. The consequences feared by the participants were abandonment by their partner, loss of income and social degradation.

Combating stigma in the community, although essential, will take time. Therefore necessary steps toward encouraging HIV infected women to seek treatment include:

• Reducing self-stigma on an individual level through lengthy and repeated supportive counselling.

• Assisting them to form empowering relationships through post-test peer groups that convene in settings other than the CTC - as in the case of the FAUMA club at a nearby school, or at the RCH clinic if facilities there are available.

• Emphasizing by example the beneficial effect of treatment for themselves and for their children through encounters with other PLHIV who are already receiving treatment.

• Supporting the women being referred to the CTC during their first visit by escorting them to the clinic and remaining there with them, until they feel comfortable or have been linked to supportive staff employed or volunteering at the CTC.

• Placing the CTC and in particular its entrance in a discrete location to further increase its accessibility.

• Further studies to assess whether helping HIV infected women to gain financial independence through support of income generating initiatives would promote independence and decrease fear of abandonment and subsequently lead to increased uptake of HAART.

## Competing interests

The authors declare that they have no competing interests.

## Authors' contributions

The study was conceived and designed by ZPT, BPT, TLK and JG withparticipation from MGC, ICB and MML. Interviews were conducted by ZPT andCP. Focus group discussions were conducted by ZPT and MGC. Data-analysiswas done by ZPT with guidance from BPT. The manuscript was drafted by ZPT, TLK and BPT and underwent critical reading by MGC, CP, ICB, JG and MML. Allauthors have approved the final manuscript.
